# Socio-economic, demographic and geographic correlates of cigarette smoking among Indonesian adolescents: results from the 2013 Indonesian Basic Health Research (RISKESDAS) survey

**DOI:** 10.1080/16549716.2018.1467605

**Published:** 2018-06-01

**Authors:** Nunik Kusumawardani, Ingan Tarigan, Suparmi, Anne Schlotheuber

**Affiliations:** a National Institute of Health Research and Development (NIHRD), MoH, Jakarta, Indonesia; b World Health Organization, Geneva, Switzerland

**Keywords:** Indonesia, adolescents, smoking, health inequality, health equity

## Abstract

**Background**: The prevalence of adolescent tobacco use in Indonesia is among the highest in the world. Monitoring the extent and distribution of adolescent cigarette smoking is crucial to being able to target prevention and reduction strategies and evaluate the effectiveness of interventions.

**Objectives**: To quantify the prevalence of adolescent cigarette smoking in Indonesia and assess the association with key socio-economic, demographic and geographic factors.

**Methods**: We used data from the 2013 Indonesian Basic Health Research (RISKESDAS) national household survey to quantify the prevalence of cigarette smoking in adolescents aged 10–18 years by sex, age, education, economic status, place of residence and province. We used logistic regression to assess the adjusted association between adolescent smoking and these factors.

**Results**: The overall smoking prevalence among Indonesian  adolescents was 7.2% (95% Confidence Interval/CI: 7.1–7.4). The prevalence was substantially higher among males (14.0%; 95% CI: 13.6–14.4) compared with females (0.2%; 95% CI: 0.1–0.4). After controlling for socio-economic, demographic and geographic characteristics, higher odds of smoking were observed among males (OR = 118.1; 95% CI: 91.2–153.0) as compared to female and among  adolescents aged 13–15 and 16–18 years as compared to those aged 10–12 years (OR = 13.2; 95% CI: 10.8–16.2 and OR = 72.7; 95% CI: 59.1–89.4, respectively). The odds of smoking were greater among adolescents with higher education as compared to those with lower education (OR = 1.3; 95% CI: 1.1–1.4) and adolescents in the poorest quintile had more than twice the odds of smoking compared with adolescents from the richest quintile (OR = 2.5; 95% CI: 2.2–2.8).

**Conclusion**: Smoking prevention and cessation interventions in Indonesia need to be specific considering the sex, age, socioeconomic status and geographic location of adolescents. Ongoing monitoring of adolescent smoking is important for targeting interventions at higher-risk groups and assessing the effectiveness of current tobacco control strategies.

## Background

The negative consequences of smoking are well documented globally; they include overall diminished health and diseases of almost all the organs as well as harm to both mother and foetus when mothers smoke during pregnancy [1,2]. The effects of second-hand smoke are also considerable: sudden infant death syndrome, middle-ear diseases, respiratory diseases, coronary heart diseases, stroke and lung cancer across genders, as well as impacts on the reproductive health of women [3,4]. The direct and indirect costs of smoking, in terms of health service use for diseases attributable to smoking and productivity loss from smoking-related morbidity and mortality, represent a significant burden to individuals, families and societies [1,2,4]. This burden is especially pronounced in low- and middle-income countries, exacerbating impoverishment and hindering social and economic development [5].

The impacts are severely felt in low- and middle-income countries particularly since they now have the greatest prevalence of smoking [6,7], and tobacco companies tend to be under-regulated in most of these countries [6]. Cigarette smoking and addiction are most likely to begin during adolescence (before 18 years of age) [7–10]. Evidence showing the addictive nature of nicotine as well as the harm adolescent exposure causes to brain development provides impetus for tobacco control measures aimed at preventing the early initiation of smoking [11].

Indonesia has among the highest global prevalence rates of cigarette smoking in the world: in 2011, about 33% of individuals aged 15 years and above smoked daily [11]. The Indonesia 2014 Global Youth Tobacco Survey (GYTS), a nationally representative school-based student survey, reported a prevalence of 18.3% of ‘current cigarette smokers’ in the 13–15 year age group; 33.9% of all boys surveyed reported current cigarette smoking compared with 2.5% of all girls surveyed [12]. Results from a report of the Indonesian Ministry of Health showed that the prevalence of smoking among adolescents aged 10–18 years (both in and out of school) was 7.2% in 2013 [13].

The association between cigarette smoking and socio-demographic factors has been reported for a range of different settings (high-, middle- and low-income countries) and population subgroups, including adults and adolescents. An analysis of socio-demographic determinants of tobacco use in six Southeast Asian countries showed that the highest prevalence of male smoking was in Indonesia (76.4%) and the highest prevalence of female smoking was in Nepal (15.7%); smoking prevalence was higher among the poor and uneducated [14]. A global study of adult tobacco smoking in 48 low- and middle-income countries from 2002 to 2004 showed that an overall prevalence of current smoking was higher in middle-income countries compared to the low-income countries (male: 40.7% vs. 36.1% and female: 13.2% vs. 6.2%) [15]. A study in the United States of America (USA) showed that current cigarette use increased by educational level and age: cigarette use among grade 12 students was about 2.5 times that of grade 8 students, while the Odds Ratio (OR) of current cigarette use was 1.59 for students aged 14 years and above as compared to those aged 13 years and younger [16].

Results from the Indonesia 2012 Demographic and Health Survey (DHS) showed similar results: smoking prevalence was higher in older age groups of 20–24 years (68.1%) as compared to younger adolescents aged 15–19 years (43.3%). Adolescents with less than primary schooling had greater smoking prevalence (71.4%) than those who had completed secondary or higher education (53.8%) [17]. Urban rural differences were minor: smoking prevalence among male adolescents (age 15 to 24 years) was marginally higher in rural (53.7%) than in urban (51.9%) populations.

While socio-economic, demographic and geographic inequalities in cigarette smoking have been reported for adults in Indonesia, there is limited published research on such associations among Indonesian adolescents – both in and out of school – at the provincial level in Indonesia. An example of such work is the 2014 GYTS for Indonesia, which reported findings by sex and age, but did not report the prevalence of adolescent smoking by socio-economic factors such as wealth or income or regions/provinces [12]. However, monitoring patterns of cigarette smoking among Indonesian adolescents by dimensions such as sex, age, education, economic status and geographic area may help identify and target disadvantaged subgroups for whom customized interventions may be used. On the other hand, if differences do not exist, a population based approach for all adolescents could be considered.

Against this background, the aim of this study was to use the most recently available data representative at the national and province level to quantify the prevalence of adolescent cigarette smoking in Indonesia and assess the association with key socio-economic, demographic and geographic factors.

## Methods

This study used data from the 2013 Indonesian Basic Health Research (RISKESDAS) survey. RISKESDAS is a nationally representative household health survey that has been conducted every three years since 2007 (in 2007, 2010 and 2013) by the National Institute of Health Research and Development (NIHRD), Ministry of Health [18].

In 2013, about 1,027,763 individuals from 33 provinces were interviewed as part of the RISKESDAS survey. Participants were selected using a multistage systematic random sampling method. The first stage identified groups of census blocks and designated them as primary sampling units (PSUs). The second stage used a probability proportional to enrolment size design to identify a census block from each PSU. The third stage comprised systematic random sampling of 25 census buildings from each census block. One household from each census building was randomly chosen at the fourth stage. All household members (defined as those staying in the premises for the past six months or more and having the same financial source for foods) of each selected household were asked to participate in the survey. Informed consent procedures were followed and information was collected through face-to-face interviews by trained fieldworkers using structured questionnaires. Survey participants aged under the age of 15 years were accompanied by a parent or guardian during the interview [18].

For this analysis, we restricted the sample to adolescents aged 10–18 years (N = 185,628 comprising 95,352 males and 90,276 females). The age range 10–18 years was selected because it corresponds with the school-going age-group in Indonesia as well as with the age range defined as adolescents in the Indonesian National Health Strategy 2015–2019 [19].

The dependent variable in this analysis was current cigarette smoking. Adolescents were considered to be currently smoking if they had smoked at all in the month preceding the interview. Independent variables included sex, age, education, economic status, place of residence and province. Adolescents were grouped by sex (male, female) and in 3 age groups (10–12, 13–15 and 16–18 years). Education was measured using the following four categories: did not complete elementary school, completed elementary school (grades 1 to 6), completed junior high school (grades 7 to 9) and completed or in senior school (grades 10 to 12). Economic status was measured using a wealth index that we constructed using principle component analysis (PCA), generating quintiles ranging from quintile 1 (poorest) to quintile 5 (richest). The 12 variables used for PCA – source of drinking water, type of cooking fuel, use of toilet facilities, type of toilet, behaviour of stool disposal, type of light, motor cycle ownership, TV ownership, boiling water heater ownership, 12 kg gas cylinder ownership for cooking, refrigerator ownership, and car ownership – were based on the 2010 Indonesia National Socioeconomic Survey with some modification to existing economic status related variables in 2013 RISKESDAS [18]. The PCA explored 54 models, and this set of 12 variables had the highest proportion of variation explained (53.6%) [18].

Additional independent variables were place of residence (urban vs. rural) and province (33 Indonesian provinces), used as proxies for geographic location.

All statistical analyses were conducted in Stata SE version 14, taking into account the complex survey sampling design, including stratification, cluster sampling and sample weights. The prevalence of smoking was reported overall and for population subgroups defined by socio-economic, demographic and geographic factors using weighted percentages and corresponding 95% confidence intervals (CIs). Logistic regression analysis was carried out to assess the adjusted associations between current adolescent smoking and independent variables (sex, age groups, education, economic status, place of residence and province). Adjusted odds ratios (ORs) were reported along with their 95% CIs.

## Results

In 2013, the national prevalence of current cigarette smoking among adolescents aged 10–18 years in Indonesia was 7.2%. Prevalence varied by sex, age group, place of residence, education, economic status, place of residence, and province (see Table 1).

**Table 1.** Characteristics and weighted prevalence of current cigarette smoking among Indonesian adolescents aged 10–18 years (n = 185,628) (RISKESDAS 2013).

	Sample size	Weighted prevalence of current smoking (95% CI)	P value
**National average**	185,628	7.2 (7.1–7.4)	
**Sex**			**< 0.001**
Male	95,352	14.0 (13.6–14.4)	
Female	90,276	0.2 (0.1–0.4)	
**Age**			**< 0.001**
10–12 years	69,083	0.4 (0.3–0.5)	
13–15 years	63,353	5.1 (4.8–5.4)	
16–18 years	53,195	18.9 (18.3–19.4)	
**Education**			**< 0.001**
Incomplete elementary school	63,409	2.0 (1.9–2.2)	
Complete elementary school	63,086	6.3 (6.0–6.7)	
Complete junior high school	46,762	13.3 (12.9–13.9)	
Complete senior high school or higher	12,371	16.4 (15.4–17.4)	
**Economic status**			**< 0.001**
Quintile 1	36,000	8.5 (8.0–8.9)	
Quintile 2	36,213	8.4 (8.0–8.8)	
Quintile 3	36,265	7.6 (7.2–8.1)	
Quintile 4	38,010	7.2 (6.7–7.6)	
Quintile 5	38,540	4.9 (4.6–5.3)	
**Place of residence**			**0.158**
Rural	102,934	7.4 (7.1–7.6)	
Urban	82,694	7.1 (6.8–7.4)	
**Province (by island)**			**< 0.001**
Sumatra			
Aceh	7540	7.0 (6.2–7.9)	
North Sumatra	15,270	6.1 (5.5–6.7)	
West Sumatra	7434	7.6 (6.7–8.5)	
Riau	5214	5.2 (4.4–6.0)	
Jambi	4052	6.0 (5.1–7.1)	
South Sumatera	6852	7.9 (7.1–8.8)	
Bengkulu	3465	7.4 (6.3–8.8)	
Lampung	5825	5.9 (5.1–6.8)	
Bangka Belitung	1898	5.9 (4.9–7.2)	
Riau Islands	1973	8.0 (5.5–11.3)	
Java-Bali			
DKI Jakarta	2105	6.6 (5.4–8.1)	
West Java	13,957	8.4 (7.8–9.0)	
Central Java	14,072	7.1 (6.6–7.7)	
DI Yogyakarta	1482	9.3 (7.6–11.5)	
East Java	14,416	8.2 (7.6–8.8)	
Banten	4567	8.8 (7.6–10.1)	
Bali	3096	4.2 (3.4–5.0)	
Nusa Tenggara Islands			
West Nusa Tenggara	4158	8.1 (7.2–9.2)	
East Nusa Tenggara	8880	5.4 (4.7–6.0)	
Kalimantan			
West Kalimantan	5610	5.5 (4.8–6.2)	
Central Kalimantan	4056	4.7 (3.8–5.8)	
South Kalimantan	4231	6.3 (5.4–7.3)	
East Kalimantan	4309	4.1 (3.3–5.1)	
Sulawesi			
North Sulawesi	4141	7.2 (6.2–8.4)	
Central Sulawesi	3991	7.6 (6.5–8.8)	
South Sulawesi	9115	8.2 (7.5–9.0)	
Southeast Sulawesi	4593	5.0 (4.2–5.9)	
Gorontalo	2164	8.2 (6.9–9.6)	
West Sulawesi	2153	5.8 (4.8–7.0)	
Maluku Islands			
Maluku	4078	5.9 (4.7–7.4)	
North Maluku	3145	6.3 (5.2–7.6)	
Papua			
West Papua	2234	5.1 (3.8–6.9)	
Papua	5552	5.3 (4.3–6.5)	

Note: Estimates and 95% CIs are reported as percentages.

Current cigarette smoking prevalence was substantially higher among males (14.0%) as compared to females (0.2%, p < 0.001), and among older adolescents (18.9% for 16–18-year olds) as compared to younger adolescents (0.4% for 10–12-year-olds and 5.1% for 13–15-year olds; p < 0.001). Prevalence of current cigarette smoking was lowest among adolescents with the lowest level of education and increased with increasing education levels, from 2.0% among adolescents with incomplete elementary school education to 16.4% among adolescents who had completed or were in high school (p < 0.001). There is a possibility that education was confounded by age in that older adolescents also likely had higher education. However, we noted that among those who were aged 16 to 18 years, about 1.2% had never attended school, 4.0% had not completed elementary school, 16.8% had completed elementary school, 55.6% had completed junior high school, 22.2% had completed senior high school and 0.2% had completed a diploma or college.

**Figure 1.**
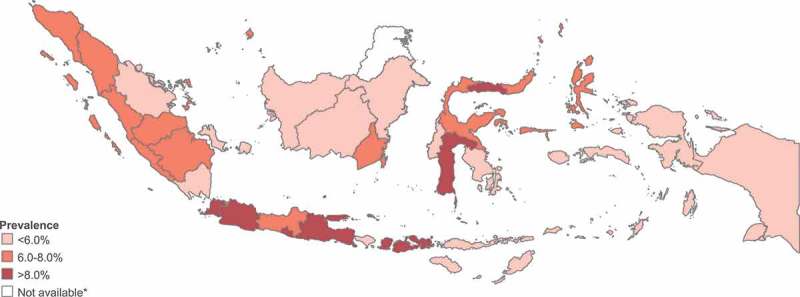
Current cigarette smoking prevalence in adolescents (age 10–18 years) in 33 provinces, Indonesia (RISKESDAS 2013).

Note: Data are not available for the province North Kalimantan, which was created in 2012.

We also found that current cigarette smoking prevalence was highest among adolescents from the poorest quintile and decreased with increasing economic status, from 8.5% in quintile 1 to 4.9% in quintile 5 (p < 0.001). There was no significant difference in current cigarette smoking prevalence between rural and urban residents (7.4% and 7.1%, respectively; p = 0.158). Current cigarette smoking prevalence varied between provinces, ranging from 4.1% in East Kalimantan to 9.3% in DI Yogyakarta. The prevalence tended to be slightly higher in the western provinces, such as Aceh (7.0%) and Bengkulu (7.4%) compared with provinces in the east such as Papua (5.3%) and West Papua (5.1%) (see Figure 1). Provinces on Java Island had some of the highest adolescent smoking rates in Indonesia.

Table 2 presents the crude and adjusted odds ratios from the logistic regression analysis. The results show that smoking remained strongly associated with sex: after adjusting for key socio-economic, demographic and geographic factors, males had more than 118 times the odds of smoking compared with females. Similarly, there remained evidence for a strong association between smoking and age, with 13–15-year-olds having more than 13 times the odds and 16–18-year-olds having more than 72 times the odds of smoking compared with 10–12-year-olds (OR = 13.24; 95% CI: 10.82–16.19 and OR = 72.68; 95% CI: 59.06–89.43, respectively). The odds of current cigarette smoking were slightly higher among adolescents with complete elementary school education or higher compared with adolescents with incomplete elementary school education. However, there was no significant difference between adolescents with completed elementary school (OR = 1.16; 95% CI: 1.05–1.30), completed junior high school (OR = 1.14; 95% CI: 1.02–1.27) or completed senior high school or higher education (OR = 1.25; 95% CI: 1.08–1.44). In addition, a co-linearity test showed no co-linearity across independent variables (Pearson Correlation< 0.8). Particularly between age groups and education, the Pearson correlation value was 0.729. A co-linearity diagnostics showed the mean Variation Inflation Factor (VIF) was 2.13 that indicated less co-linearity between age groups and education.

**Table 2.** Crude and adjusted association of current smoking among adolescents, according to socio-economic, demographic and geographic factors, in Indonesia (RISKESDAS 2013).

	Crude Odds Ratio (95% CI)	Adjusted Odds Ratio (95% CI)
**Sex**		
Male	89.5 (68.89–115.11)	118.14 (91.24–152.97)
Female	1.00	1.00
**Age**		
10–12 years	1.00	1.00
13–15 years	13.54 (11.22–16.34)	13.24 (10.82–16.19)
16–18 years	58.96 (48.96–70.99)	72.68 (59.06–89.43)
**Education**		
Incomplete elementary school	1.00	1.00
Complete elementary school	3.31 (3.02–3.63)	1.16 (1.05–1.30)
Complete junior high school	7.54 (6.91–8.23)	1.14 (1.02–1.27)
Complete senior high school or higher	9.58 (8.59–10.67)	1.25 (1.08–1.44)
**Economic status**		
Quintile 1	1.78 (1.63–1.96)	2.49 (2.20–2.81)
Quintile 2	1.77 (1.61–1.94)	2.26 (2.02–2.53)
Quintile 3	1.59 (1.45–1.75)	1.71 (1.53–1.91)
Quintile 4	1.49 (1.35–1.64)	1.60 (1.43–1.79)
Quintile 5	1.00	1.00
**Place of residence**		
Rural	1.04 (0.98–1.11)	0.99 (0.91–1.06)
Urban	1.00	1.00
**Province (by island)**		
Sumatra		
Aceh	1.36 (1.06–1.74)	2.01 (1.50–2.69)
North Sumatra	1.16 (0.91–1.47)	1.51 (1.14–1.99)
West Sumatra	1.47 (1.15–1.87)	1.96 (1.47–2.61)
Riau	0.97 (0.75–1.27)	1.33 (0.97–1.82)
Jambi	1.14 (0.87–1.50)	1.45 (1.04–2.00)
South Sumatera	1.54 (1.21–1.96)	1.93 (1.45–2.58)
Bengkulu	1.44 (1.09–1.90)	1.88 (1.35–2.62)
Lampung	1.13 (0.87–1.46)	1.38 (1.02–1.87)
Bangka Belitung	1.13 (0.84–1.53)	1.91 (1.33–2.74)
Riau Islands	1.55 (0.99–2.41)	2.40 (1.38–4.17)
Java-Bali		
DKI Jakarta	1.27 (0.94–1.72)	2.43 (1.70–3.47)
West Java	1.63 (1.30–2.05)	2.38 (1.82–3.12)
Central Java	1.37 (1.09–1.71)	1.82 (1.39–2.39)
DI Yogyakarta	1.84 (1.35–2.52)	2.60 (1.82–3.72)
East Java	1.59 (1.27–1.99)	2.20 (1.69–2.88)
Banten	1.72 (1.33–2.23)	2.44 (1.79–3.31)
Bali	0.78 (0.58–1.04)	1.08 (0.77–1.51)
Nusa Tenggara Islands		
West Nusa Tenggara	1.58 (1.23–2.04)	2.02 (1.50–2.73)
East Nusa Tenggara	1.01 (0.79–1.29)	1.14 (0.85–1.52)
Kalimantan		
West Kalimantan	1.03 (0.80–1.33)	1.26 (0.94–1.68)
Central Kalimantan	0.89 (0.66–1.21)	1.16 (0.82–1.65)
South Kalimantan	1.20 (0.92–1.57)	1.67 (1.22–2.27)
East Kalimantan	0.77 (0.57–1.04)	1.19 (0.83–1.70)
Sulawesi		
North Sulawesi	1.40 (1.07–1.82)	1.88 (1.39–2.56)
Central Sulawesi	1.47 (1.13–1.92)	2.16 (1.56–3.00)
South Sulawesi	1.60 (1.26–2.02)	2.74 (2.07–3.63)
Southeast Sulawesi	0.93 (0.71–1.24)	1.15 (0.84–1.57)
Gorontalo	1.59 (1.21–2.10)	2.33 (1.66–3.27)
West Sulawesi	1.09 (0.82–1.47)	1.42 (1.00–2.02)
Maluku Islands		
Maluku	1.13 (0.82–1.55)	1.29 (0.90–1.85)
North Maluku	1.20 (0.90–1.60)	1.44 (1.02–2.02)
Papua		
West Papua	0.97 (0.66–1.41)	1.23 (0.79–1.92)
Papua	1.00	1.00

Note; The adjusted odds ratio are adjusted for sex, age, economic status, education, place of residence and province.

Adolescents from the two poorest quintiles had more than double the odds of current cigarette smoking than adolescents from the wealthiest quintile (Quintile 1 OR = 2.49; 95% CI: 2.20–2.81; Quintile 2 OR = 2.26; 95% CI: 2.02–2.53), while adolescents from quintiles 3 and 4 had more than 1.5 times the odds of smoking compared with adolescents from the richest quintile (Quintile 3 OR = 1.71; 95% CI: 1.53–1.9; Quintile 4 OR = 1.60; 95% CI: 1.43–1.79). There was no statistically significant difference between adolescents from quintiles 1 and 2 and between adolescents from quintiles 3 and 4, respectively. There was also no statistically significant association between smoking and place of residence (OR = 0.99; 95% CI: 0.91–1.06). Adolescents residing in the provinces of DI Yogyakarta and South Sulawesi had more than 2.5 times the odds of smoking compared with their counterparts in the province of Papua, after controlling for individual socio-economic and demographic characteristics (OR = 2.60; 95% CI: 1.82–3.72 and OR = 2.74; 95% CI: 2.07–3.63, respectively). The odds of smoking tended to be higher for adolescents living in western provinces compared with adolescents living in the eastern part of Indonesia.

## Discussion

This study addresses an important gap in the literature by quantifying the extent and distribution of current cigarette smoking among Indonesian adolescents aged 10–18 years by key socio-economic, demographic and geographic factors, using nationally representative data. We found that that the current cigarette smoking prevalence was significantly higher among males than females and that the prevalence increased with age. Adolescents from poorer households were more likely to smoke than those from richer households, as were adolescents living in the provinces of DI Yogyakarta and South Sulawesi. The association between education level and smoking was statistically significant, but weak. There was no significant difference in smoking prevalence between rural and urban areas.

The gender pattern observed in our study of adolescents aged 10–18 years was also observed in the 2014 Indonesia GYTS focusing on 13–15-year-olds, which identified an overall smoking prevalence of 18.3% (33.9% of boys and 2.5% of girls) [12]. Another country in the region, Malaysia, had a smoking prevalence of 11.5% among students aged 12–17 years [20]. This study found higher odds of male adolescent smoking (both crude and adjusted), in line with our findings. The Global Adult Tobacco Survey (GATS) in Egypt (2009) and China (2010) also found higher odds of smoking prevalence among men aged 15 years and above compared to women, (Egypt OR: 162.2; 95% CI: 110.9–237.3; China OR: 82.9; 95% CI: 63.7–106.0) [21].

The strong association between smoking and gender in Indonesian adolescents has been attributed in part to cultural norms against women smoking in Indonesia. Smoking is uncommon behaviour among girls and is culturally unacceptable in most part of Indonesia. Conversely, for boys, smoking has been deemed part of the social construction of masculinity [22], receiving social sanction. Tobacco consumption among males dates back to ancient Javanese ethno-medical traditions for pain management [23].

Our findings regarding the age pattern of current smoking among adolescents were consistent with the existing literature from Indonesia and elsewhere. Increasing age is associated with increasing influence of and exposure to peers and peer pressure to smoke [24–26]. The socio-economic gradient in smoking found in our study has also been seen in other studies and geographic locations. A study in Canada had shown an association between low social economic status and risk of youth smoking among native-born Canadians [7]. Surveys in several Southeast Asian countries have shown greater prevalence among those with poorer economic status [14]. Another study of smoking prevalence among students in grades 8, 10 and 12 during the years 2000 to 2006 of the Monitoring the Future (MTF) study in the USA used subsidized lunch as a proxy for low economic status: tobacco use was greater among students who received free or reduced-cost lunches, particularly among 12th graders [16].

This trend carries over to adulthood as indicated in the 2011–2012 Global Adult Tobacco Survey (GATS) across many countries [21]. Indeed, evidence suggest that it is not only wealth but also other proxies of household socio-economic status – like low parental education levels, single-parent families and low-skilled parental employment – that are all associated with higher prevalence of adolescent cigarette smoking [7,22,24].

The statistically significant but weak association between education level and smoking is likely because of the strong relationship between age and educational attainment among adolescents aged 10–18 years, in that much of the variability in the prevalence of cigarette smoking by education level is likely accounted for by age. A correlation test showed a correlation, albeit not as high as could have been expected.

The lack of a statistically significant association between place of residence and smoking is consistent with findings from the 2012 Indonesia Demographic and Health Survey (DHS) on ‘any tobacco use’ among individuals aged 15–49 years, which reported a non-significant association between tobacco use and place of residence (urban or rural) for men [14]. Access to cigarettes in both urban and rural areas is relatively high. Beside manufactured cigarettes, East and Central Java have small-scale, home-based industries that produce hand-rolled cigarettes at reasonable prices for rural communities, including for adolescents. In rural settings, cigarette smoking is culturally acceptable while health literacy on harmful effect of tobacco is limited [27]. Meanwhile, in urban areas, smoking is encouraged as part of social and peer pressure, particularly among male adolescents. Finally, cigarette advertising and marketing is ubiquitous across the country – in both rural and urban areas [28].

A previous analysis of adolescents smoking (age 15–19 years) in Indonesia showed increasing prevalence over time from 7.1% in 1995 to 20.5% in 2013 [29]. Apart from recent data on the overall prevalence of adolescent cigarette smoking in Indonesia [12], there is literature reporting prevalence rates in selected districts or provinces, but these findings are based on older data and they do not compare the situation between provinces [25,26,30]. This study found that smoking prevalence was higher among adolescents on Java Island, a pattern reported in a study of smoking among individuals aged 15 years and above from the same survey (the highest smoking prevalence was in West Java Province [32.7%] and the lowest was in Papua [21.9%]) [29], suggesting that adolescent and adult smoking patterns match. Further study can reveal the degree to which adolescent smoking may be linked to spillover effects of adult smoking (a culture of smoking, or greater likelihood of smoking initiation). These findings, reported in the 2013 survey, vary from the geographic pattern seen in 2007. At this time, the province of Bengkulu in Sumatra Island had the highest prevalence of smoking among those aged 15 and over (38.7%), while West Java Province was ranked fifth (37.1%) [29]. So accompanying the national trend of reduction in smoking prevalence between 2007 and 2013, these spillover trends appear to be a corollary trend – warranting further study.

Some insights emerge from the fact that the first tobacco industry was built in Kudus, a small city in Central Java in 1906, which produced the world-renowned Indonesian ‘clove cigarette’. The biggest tobacco industries in Indonesia are located in East and Central Java. This gives context to some of the patterns seen here, and should be further explored.

Under the World Health Organization’s Framework Convention on Tobacco Control (FCTC), the concept of MPOWER was introduced, comprising the following six policies: monitoring tobacco use and prevention policies; protecting people from tobacco smoke; offering help to quit tobacco use; warning about the dangers of tobacco; enforcing bans on tobacco advertising, promotion and sponsorship; and raising taxes on tobacco. Although Indonesia has not yet signed or ratified the FCTC, the country’s national health policy and strategy of tobacco control are guided by MPOWER. The government, in collaboration with non-government organizations (NGOs), has shown some commitment to address tobacco-related health issues. More specifically, Government Regulation 109 was instituted in 2012, which regulates cigarette production, sales and advertising and calls for nicotine information labelling on cigarette packaging, and health warnings [29]. Secondly, Indonesia also has a tobacco control road map that includes all the components of MPOWER [29]. Optimal implementation of these policies remains a challenge, requiring engagement with and action by non-health sectors and political leaders. A key area of intervention is cigarette pricing: a 2011 study using data from Indonesia found that ‘cigarette prices have a significant impact on youth cigarette demand in terms of both smoking participation and smoking intensity among smokers.’ [31]. The country should seriously consider this strategy, alongside regulation of tobacco advertising targeting young people, which has been found to be much higher in Indonesia as compared to other Association of Southeast Asian Nations (ASEAN) countries.

### Strengths and limitations

Our study has a number of strengths, Firstly, we used provincially representative data from a large household health survey. Our data likely captured greater numbers of adolescents compared with other key data sets assessing adolescent smoking, like the 2014 Indonesia GYTS, which was based on data from junior high school students (this may have underestimated adolescent smoking prevalence). RISKESDAS includes data on adolescents not in school and younger students [18]. By including adolescents aged 10–18 years, our study captured a broader age range than the 2014 Indonesia GYTS which focused on junior high school students aged 13–15 years [12]. This group is important to include, given the evidence that smoking initiation at younger ages (i.e. younger than 12 years) is associated with a greater likelihood of regular smoking in adulthood [8]. Further, our study included an analysis of adolescent smoking by province, which allowed for geographical comparisons and benchmarking, or comparison of the problem magnitude of adolescent smoking across provinces. This may help identify which provinces need stronger support and action related to tobacco control programmes in order to decrease smoking prevalence, as well in comparison to provinces with limited health resources and challenging geographic condition such as in Papua.

Limitations of our study stem from the fact that the data are cross-sectional and no inferences regarding causality may be made. Thus, while we were able to report the strength of the association between adolescent cigarette smoking and key socio-economic, demographic and geographic factors, we were unable to evaluate the causal nature of these relationships. Furthermore, the independent variables in this analysis were only those available in the 2013 RISKESDAS dataset; these were designed to measure a wider range of health indicators, and not smoking alone. Thus, other possible key determinants of adolescent smoking such as parental disapproval, affordability/availability of cigarettes, the smoking behavior of peers and older brothers (for boys), and peers’ attitudes towards smoking (for boys) [19–21,32] could not be included in the analysis. Further, we did not analyse parent–child correlations in smoking (an unpublished report of RISKESDAS data has shown an association between smoking among those aged 10–19 years and smoking among their parents [33]).

## Conclusion

Indonesia has a high prevalence of adolescent cigarette smoking as well as substantial inequalities between different subgroups. Male and older adolescents, from poorer wealth quintiles, and adolescents living in certain provinces were found to have higher odds of smoking. Tobacco control strategies need to apply specific designs based on the sex, age, economic status, and geographic location of adolescents. Targeting these subgroups with appropriate tobacco-control strategies is a key area of intervention both inside and outside schools, at individual and policy levels. The intervention strategy will be directed toward reducing prevalence and forestall initiation of smoking and will likely yield gains in reduction of smoking-related morbidity and mortality in Indonesia. At the same time, the country should consider the more upstream determinants of adolescent smoking under the MPOWER framework and, given the high prevalence, seek to introduce control policies that are more upstream, starting with provinces where prevalence is higher. In addition to this, continued monitoring of the prevalence and patterns of cigarette smoking among Indonesian adolescents is important for targeting disadvantaged subgroups and assessing the effectiveness of current strategies.

## Paper context

While socio-economic, demographic and geographic inequalities in cigarette smoking have been reported for adults in Indonesia, there is limited published research on such associations among Indonesian adolescents. In this paper, we quantify the prevalence of adolescent cigarette smoking in Indonesia and assess the association with key socio-economic, demographic and geographic factors. Our results can be used to inform tobacco prevention and control strategies to target high-risk groups and assess the effectiveness of current strategies.
